# Epithelial Cells Derived from Human Embryonic Stem Cells Display P16^INK4A^ Senescence, Hypermotility, and Differentiation Properties Shared by Many P63^+^ Somatic Cell Types

**DOI:** 10.1002/stem.64

**Published:** 2009-06

**Authors:** Sally Dabelsteen, Paula Hercule, Patricia Barron, Meghan Rice, Gregory Dorsainville, James G Rheinwald

**Affiliations:** 1 Department of Dermatology and Harvard Skin Disease Research Center, Brigham and Women's Hospital and Harvard Medical SchoolBoston, Massachusetts, USA; 2 Department of Oral Medicine, Pathology and Anatomy, School of Dentistry, University of CopenhagenDenmark

**Keywords:** Human embryonic stem, cells, Keratinocyte, Urothelial, Tracheobronchial, p63, Cell culture

## Abstract

Human embryonic stem (hES) cells can generate cells expressing p63, K14, and involucrin, which have been proposed to be keratinocytes. Although these hES-derived, keratinocyte-like (hESderK) cells form epithelioid colonies when cultured in a fibroblast feeder system optimal for normal tissue-derived keratinocytes, they have a very short replicative lifespan unless engineered to express HPV16 E6E7. We report here that hESderK cells undergo senescence associated with p16^INK4A^ expression, unrelated to telomere status. Transduction to express bmi1, a repressor of the p16^INK4A^/p14^ARF^ locus, conferred upon hESderK cells and keratinocytes a substantially extended lifespan. When exposed to transforming growth factor beta or to an incompletely processed form of Laminin-332, three lifespan-extended or immortalized hESderK lines that we studied became directionally hypermotile, a wound healing and invasion response previously characterized in keratinocytes. In organotypic culture, hESderK cells stratified and expressed involucrin and K10, as do epidermal keratinocytes in vivo. However, their growth requirements were less stringent than keratinocytes. We then extended the comparison to endoderm-derived, p63^+^/K14^+^ urothelial and tracheobronchial epithelial cells. Primary and immortalized lines of these cell types had growth requirements and hypermotility responses similar to keratinocytes and bmi1 expression facilitated their immortalization by engineering to express the catalytic subunit of telomerase (TERT). In organotypic culture, they stratified and exhibited squamous metaplasia, expressing involucrin and K10. Thus, hESderK cells proved to be distinct from all three normal p63^+^ cell types tested. These results indicate that hESderK cells cannot be identified conclusively as keratinocytes or even as ectodermal cells, but may represent an incomplete form of, or deviation from, normal p63^+^ lineage development.

## INTRODUCTION

Since their initial cultivation and characterization [1, 2], human embryonic stem (hES) cell lines have attracted interest for their potential to produce functional somatic cells for cell replacement therapy and to elucidate mechanisms of lineage development [3]. However, few reports have described derivation from hES cells of even modestly proliferative, partially purified populations displaying features of a specific somatic cell type [4–7]. A major obstacle to evaluating normality, quality, and suitability of such cells for human use is the absence of optimized culture media for many normal cell types, necessary to evaluate proliferative and differentiation potential of hES-derived cells against proper standards. Some cell types, such as epidermal keratinocytes, can be expanded greatly in culture from small biopsies from the intended recipient, obviating the necessity to derive them from hES cells for therapy. Yet, this substantial proliferative potential of postnatal epidermal keratinocytes in culture and their ability to undergo histogenesis in experimental transplant [8–10] and organotypic culture [11–13] models and to re-establish normal, functioning, and permanent tissue in autologous transplants [14–16] provides a strong rationale for generating keratinocytes from hES cells to obtain proof-of-principal for hES-derived cell therapy.

The keratinocyte is the cell type that forms stratified squamous epithelia, including the epidermis and corneal, esophageal, oropharyngeal, vaginal, and exocervical epithelia. These epithelia have distinctive cytokeratin expression patterns and suprabasal architecture [17–19] and are formed by intrinsically specialized keratinocyte “subtypes.” These are programmed during development to preferentially undergo a site-specific pattern of suprabasal differentiation and they maintain their identity during serial culture [9, 10, 13]. Human keratinocytes grow rapidly and for a long, finite replicative lifespan (25 to ∼80 population doublings [PD], varying by tissue site and donor) in optimized media [10, 20–22].

As for all normal somatic cell types, keratinocyte replicative potential in culture is subject to limitation by a p53-/ p21^cip1^-dependent senescence mechanism triggered by progressive telomere erosion. However, keratinocyte lifespan typically is determined by the timing of derepression of the *CDKN2A* gene, encoding the cell cycle inhibitors p16^INK4A^ and p14^ARF^. This event, referred to as “p16 senescence,” occurs abruptly and with ever increasing frequency during serial passage [20, 23], independent of telomere status. Keratinocytes must undergo mutations or be engineered to evade this mechanism to become immortalized by telomerase catalytic subunit (TERT) expression [20, 23–25]. p16 expression also limits replicative potential of mammary epithelial and urothelial cells and acts as a barrier to their immortalization [26, 27]. In vivo, p16 plays no role in normal epithelial tissue homeostasis or development [28, 29] but is expressed specifically by cells that become migratory at the edges of wounds or that are poised to begin invasion in premalignant lesions [30, 31]. A hypermotility response associated with growth arrest can be elicited in normal keratinocytes in an experimental culture model of this mechanism [30].

Following identification of cells expressing the cornified envelope protein involucrin and K10 in outgrowth cultures of embryoid bodies formed by murine ES cells [32], several labs identified p63^+^/K14^+^ epithelial cells with the potential to express involucrin arising from hES cells [33–37]. The transcription factor p63 and the cytokeratin protein K14 were first identified as markers and studied most intensively for their roles in epidermal development and function, but they are expressed by a variety of epithelial tissues and cell types [17–19, 38–42]. Occasional appearance of involucrin^+^ cells in hES-derived cultures led to a preliminary conclusion that these cells are keratinocytes, and we refer to them here as “hES-derived, keratinocyte-like (hESderK)” cells. hESderK cells form colonies in the feeder/FAD system [33–37] but their replicative potential is extremely limited (<15 PD), preventing their analysis. In a recent collaborative study [35], after finding that they were not immortalized by TERT transduction, we immortalized hESderK cells with HPV16 E6E7, a method first used to immortalize normal keratinocytes [43–46]. hESderK/E6E7 cells differed morphologically from normal somatic keratinocytes and underwent very limited stratification and involucrin expression [35] and we considered the possibility that these differences resulted from expression of the E6E7 oncoproteins.

Here, we report a comprehensive analysis of three hESderK lines for morphology, marker expression, growth requirements, motility, and histogenic potential, comparing them with normal primary and experimentally immortalized epidermal keratinocytes and two other p63^+^/K14^+^ epithelial cell types—urothelial and tracheobronchial epithelial cells. We evaluated effects on cell behavior of expressing bmi1, a polycomb protein that maintains repression of the p16^INK4A^/p14^ARF^ locus [47–49], to confer extended lifespan. We find that hESderK cells share many features with keratinocytes and other p63^+^ cell types but that they clearly differ from all of them, suggesting that hESderK cells are an incomplete or abnormal form of p63^+^ lineage development.

## MATERIALS AND METHODS

### Cell Lines and Culture Media

Cell lines studied are listed in Table 1. Strain N, J4Ep, HBL-10U, LP9, R2F, and TrBEp-1 are primary cell lines (also known as cell strains), that is, cells cultured from normal tissues and serially propagable for a finite replicative lifespan, during which they remain genetically and functionally normal. The p63^+^/K14^+^ “hESderK” cell population [34] derived from a teratoma formed by H9 hES cells and the hESderK/E6E7 cl2 (nod3-c2) line [35] derived from these hESderK cells were reported previously. hESderK/E6E7 clK was clonally isolated from the same mass hESderK/E6E7 population from which cl2 had been isolated. hESderK/bmi1 is a bmi1/puro-transduced cell line derived from the same original hESderK cell population [34] that had been used to generate the E6E7 clones.

**Table 1:** Cell lines studied

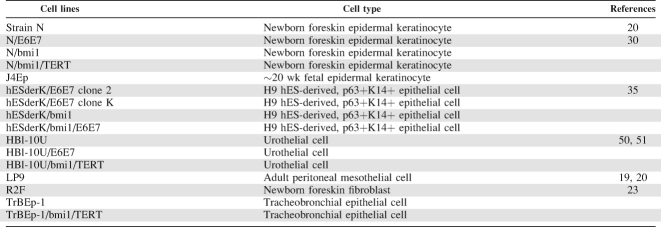

For some experiments, cells were cultured using the feeder/FAD system [20, 21, 52]: cocultivation with γ-irradiated 3T3J2 cells in FAD medium: Dulbecco's modified Eagle's medium (DMEM)/F12 (1:1 v/v) (Gibco, Grand Island, NY, http://www.invitrogen.com/Invitrogen, Carlsbad, CA, http://www.invitrogen.com) +5% iron-supplemented bovine calf serum (HyClone, Logan, UT, http://www.hyclone.com), 10 ng/ml epidermal growth factor (EGF), 0.4 μg/ml hydrocortisone, 1.8 × 10^−4^ M adenine, 10^−10^ M cholera toxin, 5 μg/ml insulin, 2 × 10^−11^ M triiodothyronine, and penicillin/streptomycin (pen/strep). For most experiments, keratinocytes and urothelial cells were cultured in keratinocyte serum-free medium (K-sfm) (Gibco) supplemented as described [20] with 25 μg/ml bovine pituitary extract (BPE), 0.2 ng/ml EGF, an additional 0.3 mM CaCl_2_ to bring total [Ca^2+^] to 0.4 mM, and pen/strep. To generate healthy high-density cultures, cells were grown to ∼40% confluence in K-sfm, then refed daily with a 1:1 (vol:vol) mixture of K-sfm and “DF-K medium,” the latter consisting of a 1:1 (vol:vol) mixture of calcium-free, glutamine-free DMEM (Gibco) and Ham's F-12 (Gibco) + 0.2 ng/ml EGF + 25 μg/ml BPE + 1.5 mM L-glutamine + pen/strep.

The primary human tracheobronchial epithelial cell line TrBEp-1 (obtained from Cambrex, Walkersville, MD, http://www.cambrex.com) and its derivatives were cultured in BEGM medium (Cambrex). R2F primary human fibroblasts and LP9 primary human mesothelial cells were grown in serum- and mitogen-supplemented media as described [20, 23].

To compare growth in different media, 9 cm^2^ wells were plated with 2,000 cells, grown for 7–9 days with refeeding every 2 days, and then trypsinized and counted. Growth rates were calculated as log_2_(no. cells counted/no. cells plated)/no. days = PD/day.

For replicative lifespan determination, cells were plated at 10^4^ to 10^5^ cells/p60 or p100 dish, refed every 2–3 days, and subcultured 5–9 days after plating before growth was slowed by density. PD was calculated as log_2_(no. cells at subculture/no. cells plated). Cumulative PD was plotted against total time in culture to determine onset of senescence or immortalization [20, 23].

### Organotypic Culture

Organotypic cultures were prepared as described [12, 13, 23]. Foreskin fibroblasts (3 × 10^5^) suspended in 3 ml of bovine collagen I (1.1 mg/ml) (Organogenesis, Canton, MA, www.organogenesis.com) were allowed to gel over a 1 ml layer of acellular collagen in six-well culture inserts with 3-μm pore polycarbonate filters (Corning Life Sciences, Lowell, MA, http://www.corning.com/lifesciences). Gels were allowed to contract for 4–5 days before seeding with 2.5 × 10^5^ epithelial cells in serum- and mitogen-supplemented DMEM/F12 raft medium [12, 13, 23]. Inserts were raised to the air-liquid interface 4 days later and refed every 3 days for an additional 10 days. Membranes with organotypic cultures were fixed in 10% formalin, paraffin-embedded, cut as 5-μm sections, and immunostained. Two experiments conducted at different times with each cell line yielded organotypic cultures with similar morphologies. Measurements at 10 points across the central ∼5 mm cross section of each organotypic culture yielded SEM values ≤8% for thickness and numbers of total and stratum corneum cell layers.

### Retroviral Vectors and Transduction

BABE-bmi1.puro, Wzl-TERT.Bsd, and L(E6E7)SN vector plasmids were transfected into Phoenix or PA317 cells to produce amphotropic packaging cells, which were incubated for 8 hours in 1:1 medium 2–4 days after transfection to obtain retroviral supernatants. As described [13, 20], epithelial cells plated 1–2 days earlier at ∼10^5^ cells per 9 cm^2^ well were transduced for ∼6 hours with retroviral supernatant +2 μg/ml polybrene (Sigma-Aldrich, St. Louis, http://www.sigmaaldrich.com). Transduced cells were subcultured the next day into p100 dishes with K-sfm medium, or p60 dishes with feeder cells and FAD medium. Drug selection (0.5 μg/ml puromycin, 5 μg/ml blasticidin, or 0.2 mg/ml G418) began 1 day after transduction and continued for 5–7 days. Some transductants were made by coplating cells with γ-irradiated retroviral producer cells in FAD medium. Producer cells were selectively removed 3–4 days later by brief incubation with trypsin/EDTA and vigorous pipetting and irradiated, drug-resistant 3T3J2 cells +drug were added back to cultures.

### Antibodies

Murine monoclonal antibodies (MuMoAb) used were as follows: p16^INK4A^ (G175-405 [BD Pharmingen, San Diego, CA, http://www.bdbiosciences.com/index[lowen]us.shtml]); Laminin γ2 chain (D4B5 [Chemicon, Temecula, CA, http://www.chemicon.com]); bmi1 (clone F6 [Millipore, Billerica, MA, http://www.millipore.com]); involucrin (SY5 [Research Diagnostics, Flanders, NJ, http://www.researchd.com]); K14 (CKB1 [Sigma-Aldrich]); p63 (4A4 [provided by F. McKeon, Harvard Medical School]); and K10 (AE20) and K5 (AE14) (provided by T.T. Sun, N.Y.U. Medical School).

### Immunocytochemistry and Immunohistochemistry

Cultured cells were fixed and permeabilized in 100% cold methanol, or fixed in fresh 4% paraformaldehyde and permeabilized with Triton X-100, and immunostained using avidin-biotin-complex (ABC) peroxidase and NovaRed substrate (Vectastain Elite ABC, Vector Laboratories, Burlingame, CA, http://www.vectorlabs.com), as described [30, 31]. Five-micrometer paraffin sections of organotypic cultures were immunostained the same way. Antigen retrieval, by microwaving slides submerged in citrate buffer (pH 6), was used before immunostaining sections for p63. Images were captured on a NIKON E600 Microscope with a SPOT2 digital camera using SPOTcam v.3.5.5 software (Digital Instruments, Tonowanda, NY, http://www.digitalinstruments.com).

### Hypermotility Assays

Transforming growth factor beta (TGFβ)- and Lam-332′-induced hypermotility was visualized and quantitated as described [30]. Conditioned medium (CM) from the rat bladder carcinoma cell line 804G [53] was used as a source of γ2 chain-unprocessed Laminin-332 (Lam-332′) [30, 54]. 804G cells were grown to confluence in DMEM + 10% calf serum, refed with fresh medium, and returned to the incubator for 8–16 hours. The CM was 0.2-μm filter sterilized (Nalge/Nunc, Rochester, NY, http://www.nalgenunc.com) and stored at −20°C. Wells (9 cm^2^; Corning Life Sciences) were precoated by incubating with 804G CM diluted one-third with 10% serum DMEM for 30 minutes at 37°C and rinsed 3× with phosphate-buffered saline (PBS) and once with K-sfm before plating cells in K-sfm. For some experiments, cells were plated on untreated dishes in K-sfm +0.3 ng/ml recombinant human TGFβ1 (R&D Systems, Minneapolis, MN, http://www.rndsystems.com), added from a 1,000× concentrated solution in 4 mM HCl +0.1% BSA.

To measure directional hypermotility, wells plated with 700 cells were fixed 1 day after plating on Lam-332′-precoated dishes, or 2 days after plating in 0.1 mM Ca^2+^ K-sfm +TGFβ, in 10% formalin/PBS for 30 minutes and immunostained as described earlier with Laminin γ2 MuMoAb D4B5 (Chemicon). Fifteen to 20 fields were photographed using a 2× objective. Laminin-332 pads and tracks deposited by >100 cells were scored for length and width, as described [30]. Cells that deposited Laminin-332 tracks with <4:1 asymmetry were classified as nonmotile, with tracks of 4:1-10:1 asymmetry and <500 μm in length as slightly motile, and with tracks >500 μm in length as hypermotile.

### Western Blotting

Proliferating cultures were trypsinized, PBS-rinsed, lysed in 20 mM Tris buffer (pH 7.3) +2% SDS +protease inhibitor cocktail (Roche Diagnostics, Indianapolis, IN, http://www.roche-applied-science.com) and sonically disrupted. Thirty micrograms (Bio-Rad assay) of extract protein was separated by SDS-PAGE under reducing conditions in precast, 4%–20% gradient gels (Bio-Rad, Hercules, CA, http://www.bio-rad.com), electrotransferred to PVDF membranes (Millipore), and specific proteins detected by incubation with primary antibodies, peroxidase-conjugated secondary antibody (Southern Biotech, Birmingham, AL), and ECL chemiluminescence reagent (Amersham, Buckinghamshire, UK, http://www.amersham.com). Membranes were stripped of antibody using BlotFresh (SignaGen Labs, Gaithersburg, MD, http://www.signagen.com) and reprobed with β-actin antibody (A-2066; Sigma) as loading control.

## RESULTS

### The Replicative Potential of hESderK Cells Is Limited by a p16^INK4A^-Enforced Senescence Mechanism

**Figure 1:**
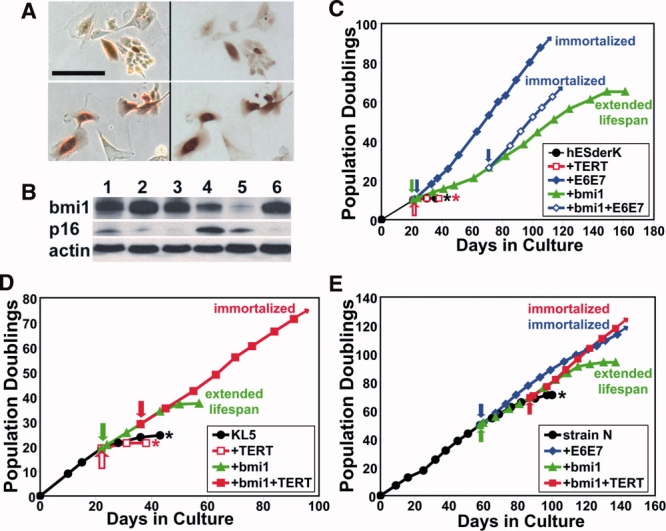
Extending the replicative lifespan of hES-derived epithelial cells by evading the p16^INK4A^ senescence mechanism. **(A):** p16 immunocytochemical staining of hESderK cells in their third and final passage before senescence, growing in Gibco K-sfm medium (phase contrast left; bright-field, right). Note that small proliferative, colony-forming cells are p16-negative and large, nondividing cells are p16-positive. The large, very flat cells, p16-negative in these fields are irradiated 3T3 fibroblast feeder cells that had been carried over from the previous passage. Bar in top left panel is 200 μm long. **(B):** Western blot analysis of bmi1 and p16 expression. Lanes: (1) early passage strain N epidermal keratinocyte; (2) late passage N/bmi1; (3) hESderK/bmi1; (4) hESderK/E6E7 clone K; (5) late passage HBl-10U; (6) late passage HBL-10U/bmi1. Note that constitutive bmi1 expression is associated with reduced p16 expression in normal keratinocytes, hESderK cells, and normal urothelial cells and that hESderK/E6E7 cells express high levels of p16 but are still able to proliferate, consistent with Rb-inactivation by E7. (**C–E):** Replicative lifespans of primary cell lines and engineered derivatives; arrows indicate timing of transductions during serial passage of this line; asterisks indicate the normal replicative lifespan limit (senescence) of the primary cell line. **(C):** Lifespan extension and immortalization of hESderK cells. Note that hESderK cells senesced by 10 population doublings (PD) and were not immortalized by TERT. Expression of bmi1 in the parent line conferred a long extension of lifespan to ∼60 PD. Transduction of the parent line to express E6 and E7 resulted in immortalization and also a substantial increase in growth rate to the ∼1 PD/day rate exhibited by early passage normal keratinocytes and their engineered derivatives. **(D):** Lifespan extension and immortalization of normal primary corneal keratinocyte line KL5. Note that KL5 had a very short lifespan of ∼23 PD, which was not altered by TERT expression. Transduction of KL5 to express bmi1 resulted in extension of lifespan to ∼37 PD and subsequent transduction of KL5/bmi1 cells to express TERT resulted in immortalization. **(E):** Lifespan extension and immortalization of normal primary human epidermal keratinocyte strain N. Note that transduction of strain N to express bmi1 resulted in extension of replicative lifespan from 70 to 90 PD, and subsequent transduction of N/bmi1 to express TERT resulted in immortalization. Transduction of strain N to express HPV16E6 and E7 also resulted in immortalization. Abbreviations: TERT, telomerase catalytic subunit.

As reported previously, the p63^+^/K14^+^ (hESderK) cell population recovered from H9 hES teratomas by cultivation in semidefined keratinocyte media or with 3T3 feeder support could not be propagated beyond 10–15 PD [35, 37]. On the basis of our experience with normal primary human keratinocytes [20], we hypothesized that hESderK cells were senescing from p16^INK4A^ induction. In their final passage, all large, nondividing hESderK cells proved to be p16^+^ (Fig. 1A). After E6E7-transduction, hESderK cells divided rapidly and indefinitely (Fig. 1C) despite p16 expression (Fig. 1B), as was also the case for E6E7-transduced epidermal keratinocytes (Fig. 1E). This was expected, considering that E7 inactivates retinoblastoma protein and renders cells indifferent to p16 expression. The E6E7 transductants grew as immortalized lines, as expected since, in addition to inactivating p53 to render cells indifferent to p14^ARF^ expression, E6 facilitates derepression of the endogenous *TERT* gene [55].

p16-deficient variants can arise in TERT-transduced, primary keratinocyte cell lines that have a long lifespan, such as strain N, and these variants prevail as the observed immortalized lines [20, 24, 25]. However, primary keratinocyte lines with short lifespans may not generate such variants following TERT transduction before all cells in the population have induced p16 and senesced (Fig. 1D). As an alternative to E6E7 expression, we transduced early passage hESderK cells to express bmi1, a polycomb protein that in early passage cells maintains the p16^INK4A^/p14^ARF^ locus transcriptionally inactive [48]. Transduction to constitutively express bmi1 substantially extended the lifespan of normal keratinocytes (Fig. 1D, 1E), as expected from a previous study [49]. hESderK/bmi1 transductants also displayed a greatly extended lifespan (Fig. 1C), associated with repression of p16 (Fig. 1B). Constitutive bmi1 expression was not sufficient to immortalize keratinocytes, but bmi1 transductants became immortalized when subsequently transduced to express TERT (Fig. 1C–1E). For unknown reasons, we have been unable to obtain a TERT-immortalized version of hESderK/bmi1, but hESderK/bmi1 has such a long lifespan that it could be studied in detail. We concluded that hESderK cells, like keratinocytes, possess an active p16-senescence mechanism that is much more readily activated in culture than it is in normal keratinocytes, but which can be evaded experimentally by E6E7 or bmi1 expression.

Early passage normal, bmi1-transduced, and E6E7-transduced keratinocytes grew rapidly, at a rate of nearly one PD per day. hESderK/E6E7 cells grew nearly this rapidly but hESderK/bmi1 grew more slowly, at ∼0.4 PD/day. When hESderK/bmi1 cells were transduced to express E6E7, they began proliferating at ∼1 PD/day and became immortalized. We concluded that the feeder/FAD culture system is permissive for hESderK cells but is not as optimal for their growth as it is for normal keratinocytes [21, 52]. Because E6E7 expression permitted hESderK cells to divide more rapidly in the feeder/FAD system, we further concluded that E6E7 expression permits hESderK cells to bypass a requirement for a mitogen or other factor not present in the feeder/FAD system or not at optimal concentrations for hESderK cells.

### Colony Morphology, Growth Requirements, and Marker Expression of hESderK Clones

**Figure 2:**
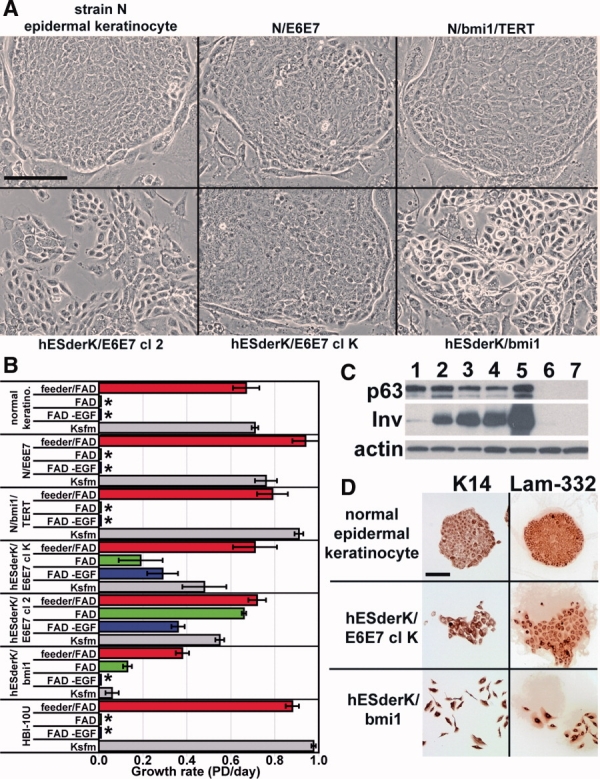
Morphology, growth, and marker expression of hESderK cells in conventional culture. **(A):** Colony morphologies in the feeder/FAD system. Shown are typical colonies formed by single cells of each line during serial passage. Note closely packed morphology with clearly demarcated cell-cell borders in colonies formed by strain N, N/E6E7, N/bmi1/TERT, and hESderK/E6E7 clone K, in contrast to the more rounded, refractile, irregular, and fragmented colony morphology of hESderK/E6E7 clone two and hESderK/bmi1. Bar in top left panel is 200 μm long. **(B):** Distinctive growth requirements displayed by hESderK cells. Data are the average of three experiments, with error bars showing standard error of the mean. Asterisks indicate conditions in which cells did not grow beyond their plating density. Note ability of the hESderK lines to grow in FAD medium without feeder fibroblasts, the absence of a stringent EGF requirement for cl2 and clK, and the poor growth of hESderK/bmi1 cells in K-sfm. Growth requirements of normal keratinocytes were unaltered by transduction to express E6E7 or bmi1+TERT. Urothelial cells (HBl-10U) displayed the same growth requirements as keratinocytes. **(C):** Western blot analysis of p63 and involucrin expression. Lanes: (1) hESderK/bmi1; (2) hESderK/E6E7 cl K; (3) hESderK/E6E7 cl 2; (4) N/E6E7; (5) strain N keratinocyte; (6) LP9 mesothelial cell; (7) R2F fibroblast. Note p63 expression by engineered hESderK and keratinocytes. Note continued expression of involucrin, albeit at reduced levels compared with normal keratinocytes, by the engineered cell lines, which correlated with relative frequencies of stratified cells in cultures of the various epithelial cell lines. **(D):** Immunocytochemical analysis of K14 and Laminin-332 expression by hESderK cells. Cells plated at low density and grown for 5 days in K-sfm medium were fixed in cold methanol and immunoperoxidase-stained for K14 and Laminin-332, revealing expression of these proteins at levels similar to that of normal epidermal keratinocytes and the deposition of secreted Laminin-332 on the culture dish surface. Bar in top left panel is 200 μm long. Abbreviations: EGF, epidermal growth factor; K-sfm, keratinocyte serum-free medium; PD, population doublings; TERT, telomerase catalytic subunit.

We compared hESderK/bmi1 and two clonally derived hESderK/E6E7 lines to normal and engineered epidermal keratinocytes. All formed colonies from single cells were serially cultivable in the fibroblast feeder/FAD system. hEsderK/E6E7 clone 2, reported previously [35], and hESderK/bmi1 both formed loosely associated, nonstratifying colonies. We isolated clone K from the initial, uncloned hESderK/E6E7-transduced population for its close-packed colony morphology resembling that of normal and E6E7- or bmi1/TERT-transduced epidermal keratinocytes (Fig. 2A).

We tested the hESderK lines for their ability to grow in several culture media permissive or nonpermissive for normal keratinocytes. Normal primary and E6E7 or bmi1+TERT transduced epidermal keratinocytes all grew well in K-sfm or in the feeder/FAD system, but could not grow in FAD medium without feeder cells (Fig. 2B). All three hESderK lines grew best in the feeder/FAD system and cl2 and clK were also able to grow in K-sfm. Notably, in contrast to normal and experimentally immortalized keratinocytes, the cl2 and clK could grow progressively in FAD medium without feeder cells, although more slowly than in the presence of feeder cells, and in medium lacking EGF. hESderK/bmi1, which grew slowly even in the feeder/FAD system, could not be propagated in K-sfm (Fig. 2B).

Western blots (Fig. 2C) of cells cultured to confluence in K-sfm medium disclosed that the engineered versions of hESderK cells and keratinocytes retained expression of p63 and involucrin. Immunohistochemical staining of preconfluent cultures of cells plated at low density in K-sfm permitted evaluation of individual cells in growing colonies for expression of these and other proteins that are expressed by normal keratinocytes. hESderK cells grown in K-sfm medium expressed K14 and the basement membrane protein Laminin-332, the latter revealed by immunostaining to be secreted onto the culture dish (Fig. 2D).

### hESderK Cells Display Hypermotility Responses to the Same Stimuli as Normal Keratinocytes

**Figure 3:**
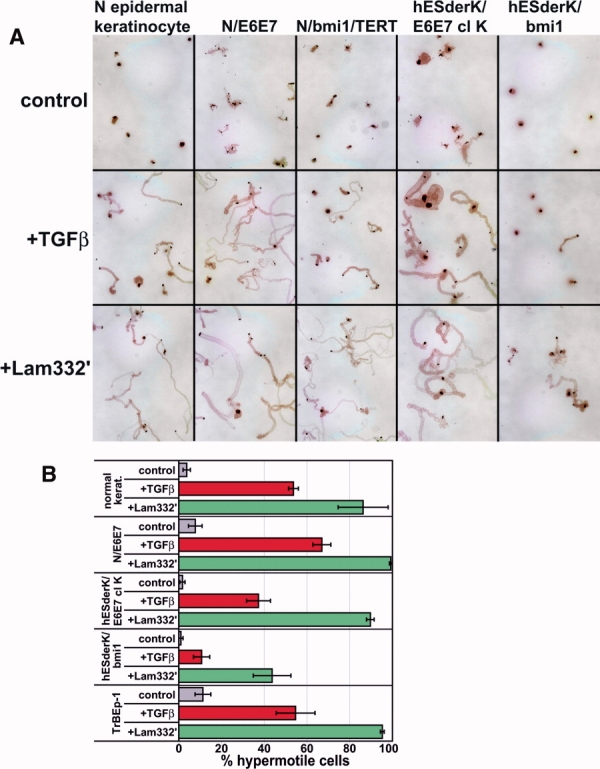
Induction of directional hypermotility in hESderK cells and normal keratinocytes. **(A):** Directional hypermotility, revealed by immunostaining for Laminin-332, comparing cells 2 days after plating in control conditions, with 0.3 ng/ml TGFβ, and plating on dishes precoated with the γ2 unprocessed form of Laminin-332 (Lam332′). Note the similar, robust motility responses of all cell lines except for hESderK/bmi1, which was responsive but weaker than the other lines. **(B):** Quantitation of hypermotility responses. Note that a smaller percentage of hESderK/bmi1 cells responded to TGFβ and Lam332′ with hypermotility than did normal epidermal keratinocytes, N/E6E7, and hESderK/E6E7 clone K cells and that normal primary TrBEp-1 were very responsive. Data are the average of three experiments, with error bars showing standard error of the mean. Abbreviations: TGFβ, transforming growth factor beta; TERT, telomerase catalytic subunit.

Our lab recently reported that normal epidermal and oral keratinocytes respond with sustained, directional hypermotility when treated with TGFβ or when plated on the γ2 chain unprocessed form of Laminin-332 (Lam332′) [30]. As revealed by tracks of Laminin-332 on the culture dish, hESderK cells also responded to TGFβ and Lam332′ with directional hypermotility (Fig. 3A). hESderK/bmi1 exhibited this response more weakly to both stimuli and hESderK/E6E7 cl K showed a somewhat reduced response to TGFβ compared with normal primary keratinocytes and their E6E7-engineered derivatives (Fig. 3B).

We concluded from all the above experiments that expression of E6E7, bmi1, or bmi1+TERT did not substantially alter the morphology, growth, or marker protein expression of normal somatic keratinocytes. Therefore, the morphologic and growth requirement differences between hESderK cells and normal keratinocytes cannot be attributed to the methods used to extend lifespan or immortalize them. Despite these differences, the expression of p63, K14, involucrin, and Laminin-332 by hESderK cells and their possession of the p16^INK4A^ senescence mechanism and the hypermotility response was consistent with an identity as keratinocyte.

### Histogenic Potential of hESderK Cells

**Figure 4:**
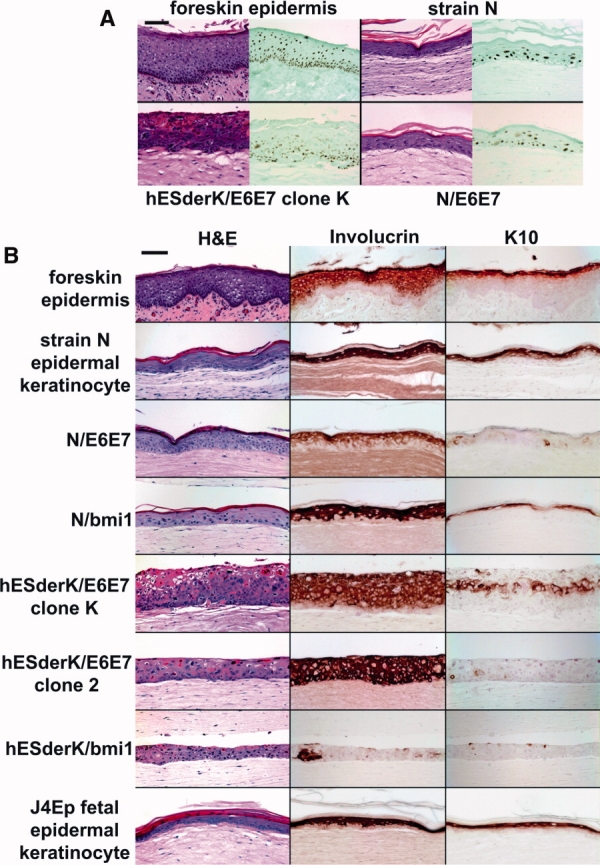
Histogenesis and differentiation-related protein expression by epidermal keratinocyte and hESderK cells in organotypic culture conditions. **(A):** Histogenesis and p63 expression (left frame hematoxylin and eosin-stained, right frame p63 immunostain with light hematoxylin counterstain), comparing foreskin tissue with organotypic cultures of strain N epidermal keratinocytes, N/E6E7, and hESderK/E6E7 clone K. Bar in top left panel is 100 μm long. **(B):** Histogenesis and involucrin and K10 expression. Panels show typical fields. Average total epithelial thickness in μm, number of cell layers, and number of stratum corneum cell layers were as follows: for foreskin epidermal tissue (100 μm, 8, 3); organotypic cultures of strain N (60 μm, 5, 2); N/E6E7 (75 μm, 5, 4); N/bmi1 (70 μm, 4, 2); hESderK/E6E7 clone K (140 μm, 10, 0); hESderK/E6E7 clone 2 (75 μm, 5, 0); hESderK/bmi1 (50 μm, 4, 0), and J4Ep (50 μm, 3, 5). Note p63 expression pattern in organotypic cultures similar to that of epidermal tissue in vivo. Note suprabasal expression of involucrin in suprabasal cells of all cell lines tested except for only sporadic expression in hESderK/bmi1 cells, and K10 expression by many suprabasal cells of strain N, N/bmi1, hESderK/E6E7 clone K, and J4Ep but only sporadic expression by N/E6E7, hESderk/E6E7 clone 2, and hESderK/bmi1. Bar in top left panel is 100 μm long.

We next compared the tissue-forming ability of hESderK cells to that of epidermal keratinocytes in organotypic culture. In these conditions, normal and E6E7-engineered keratinocytes and hESderK/E6E7 clone K cells formed stratified epithelia with a normal pattern of p63 expression (Fig. 4A). Neither E6E7 nor bmi1 expression prevented normal epidermal keratinocytes from generating a flattened, eosinophilic, stratum corneum-like upper layer (Fig. 4A, 4B). Strain N and its engineered derivatives also expressed involucrin and the epidermal differentiation protein K10 in suprabasal cells, although N/E6E7 expressed K10 only sporadically.

The three hESderK lines all formed multilayered epithelia that differed morphologically, by absence of a stratum corneum-like layer, from the epithelia formed by strain N and its derivatives and by the ∼20 wk fetal epidermal keratinocyte line J4Ep. hESderK/E6E7 clK formed the thickest epithelium (even thicker than that formed by strain N or N/E6E7) and hESderK/bmi1 the thinnest, but the morphologic structures formed by the three hESderK lines were similar (Fig. 4B). hESderK/E6E7 cl2 and clK expressed suprabasal involucrin in abundance and clK also expressed K10 in many suprabasal cells. hESderK/bmi1 expressed involucrin and K10 only in rare suprabasal cells. Thus, hESderK cells have some tissue-forming and terminal differentiation characteristics of normal epidermal keratinocytes in organotypic culture, namely, stratification and p63, involucrin, and K10 expression; yet, they differed from postnatal and from ∼20 week fetal keratinocytes by a lack of stratum corneum formation.

### Properties of Other p63^+^ Cell Types Cultured from Normal Human Epithelial Tissues

**Figure 5:**
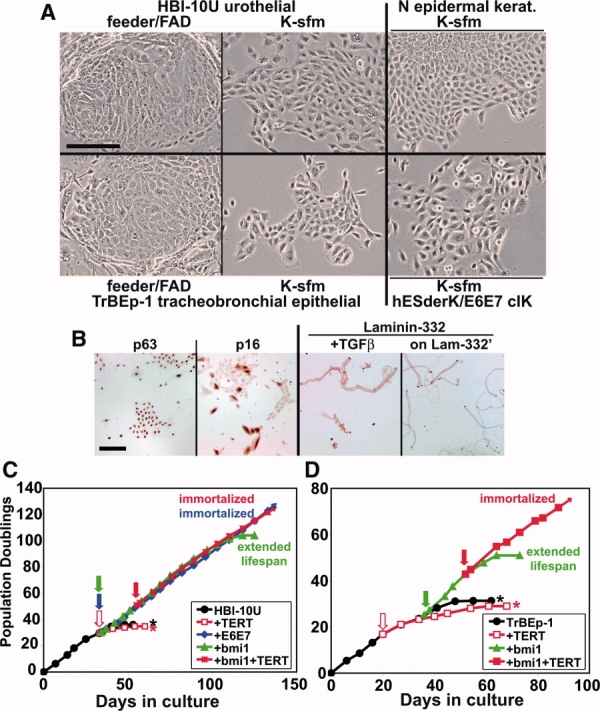
Characteristics of two nonkeratinocyte, p63+ human epithelial cell types in culture. **(A):** Colony morphologies in the feeder/FAD system and in semidefined media. Note that urothelial cells (HBl-10U) and TrBEp-1 form tightly packed colonies in the feeder/FAD system, but cell shapes are slightly different (less regular and more elongated) from that of keratinocytes (shown in Fig. 2A). Urothelial cells and tracheobronchial epithelial cells form colonies that are less cohesive than that of keratinocytes but more cohesive than that of hESderK cells in semidefined medium. (TrBEp-1 did not grow well in K-sfm, so was cultured in BEGM, as described in Methods.) Bar in top left panel is 200 μm long. **(B):** Marker expression and directional hypermotility response of urothelial cells. HBl-10U cells expressed p63 in all cells, p16^INK4A^ in large, nondividing cells, and Laminin-332, which disclosed their directional hypermotility response to TGFβ and to plating on Lam-332′. Bar in left panel is 200 μm long. **(C, D):** Immortalization of HBl-10U urothelial cells (c) and TrBEp-1 tracheobronchial epithelial cells (d), by engineering to evade the p16-senescence mechanism and to express TERT. Note that TERT expression was insufficient to immortalize TrBEp-1 cells, whereas bmi1 expression conferred extended lifespan and ability to become immortalized by subsequent transduction to express TERT. HBl-10U responded similarly to bmi1 and bmi1+TERT expression and was immortalized by E6E7. Arrows and asterisks as in Figure 2. Abbreviations: K-sfm, keratinocyte serum-free medium; TGFβ, transforming growth factor beta; TERT, telomerase catalytic subunit.

A number of epithelial cell types other than keratinocytes express p63 [41]. We examined the properties in culture of two of these: urothelial and tracheobronchial epithelial cells. Previous studies revealed that primary human urothelial cells grow well in the feeder/FAD system and maintain a distinctive pattern of cytokeratin protein expression [19, 50, 51]. HBl-10U primary urothelial cells and TrBEp-1 primary tracheobronchial cells grew well in the FAD/feeder system and formed closely packed colonies with morphologies slightly different from that of keratinocytes, in that the cells were less regular in shape and were more oval and elongated than keratinocytes (Figs. 2A and 5A). HBl-10U grew as well as keratinocytes in K-sfm (Figs. 2B and 5A) but TrBEp-1 did not (data not shown), although it did grow well in BEGM, a proprietary, BPE-supplemented medium designed for tracheobronchial cells (Fig. 5A). Urothelial and tracheobronchial epithelial cells shared with keratinocytes and hESderK cells expression of p63, p16, and Laminin-332, and hypermotility in response to TGFβ and Lam-332′ (Figs. 4B and 2D; data not shown). Both cell types proved to be subject to p16^INK4A^/p14^ARF^-enforced senescence, such that TERT transduction did not result in immortalization but transduction to express bmi1 repressed p16 expression (Fig. 1D), conferred lifespan extension, and made cells permissive for TERT immortalization (Fig. 5C, 5D).

**Figure 6:**
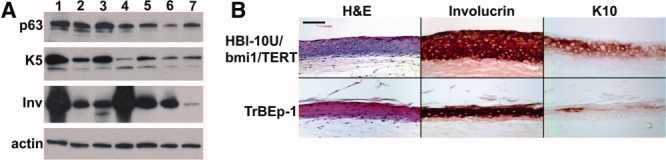
Differentiation-related protein expression in conventional culture and epithelial histogenesis in organotypic culture by urothelial and tracheobronchial epithelial cells. **(A):** Western blot analysis. Urothelial cells and tracheobronchial epithelial cells were compared with epidermal keratinocytes and hES-derived, keratinocyte-like (hESderK) cells for p63, K5, and involucrin expression during growth in the feeder/FAD culture system (except for lane 2). Lanes: (1) strain N; (2) strain N grown in keratinocyte serum-free medium medium; (3) N/bmi1; (4) HBl-10U; (5) HBl-10U/bmi1/TERT; (6) TrBEp-1/bmi1/TERT; (7) hESderK/bmi1. Note that p63 and K5 expression levels were highest for epidermal keratinocytes and were positive, but lower, for the other cell types examined. Involucrin was expressed by all cell lines, but was highest in normal epidermal keratinocytes and urothelial cells and at lower levels by bmi1 transductants of these cell types. The absence of stratification in hESderK cells cultured on plastic culture dishes likely contributed to the very low levels of this suprabasal differentiation-specific protein in hESderK/bmi1 cultures. **(B):** Suprabasal differentiation protein expression in organotypic cultures of urothelial and tracheobronchial epithelial cells. Top row: HBl-10U/bmi1/TERT. Bottom row: TrBEp-1. Panels show typical fields. Average total epithelial thickness in μm, number of cell layers, and number of stratum corneum cell layers were as follows for HBl-10U/bmi1/TERT (90μ, 6,1) and TrBEp-1 (70μ, 6, 2). Note suprabasal expression of involucrin and K10 in suprabasal cells of both cell types. Bar in top left panel is 100 μm long. Abbreviations: TERT, telomerase catalytic subunit.

We tested the normal primary somatic epithelial cell types and some of their bmi1 extended lifespan and bmi1+TERT immortalized derivatives for p63 and K5 expression (note that K5 is the partner of K14 for cytokeratin filament formation), comparing cultures grown to near confluence in the same feeder/FAD conditions to avoid the possibility that different culture medium formulations would influence protein expression. All cell types and their engineered versions retained expression of these markers (Fig. 6A). Urothelial and tracheobronchial epithelial cells both expressed involucrin in culture (Fig. 6A) which, considering their behavior in vivo, was expected for urothelial [56] but unexpected for tracheobronchial cells [57].

### Histogenic Potential of p63^+^ Epithelial Cell Types in Organotypic Culture Conditions

Finally, we examined the histogenic potential of urothelial and tracheobronchial cells (Fig. 6B). Both cell types formed multilayered epithelia with most suprabasal cells expressing involucrin, similar to normal keratinocytes and hESderK cells. K10 was expressed by most suprabasal urothelial cells but was sporadic in tracheobronchial cultures. Surprisingly, both cell types formed a thin, flattened, eosinophilic stratum corneum-like layer, unlike the tissue they normally form in vivo. Increasing the retinoic acid concentration in the medium did not suppress this (data not shown). We concluded from these experiments that p63^+^ epithelial cell types other than keratinocytes can activate expression of involucrin during growth in conventional culture, even in conditions that promote rapid proliferation, and that they can display a squamous metaplastic, keratinized form of differentiation in standard organotypic culture conditions, which had been optimized originally for epidermal keratinocyte histogenesis. This precluded conclusive identification of hESderK cells as a keratinocyte or any specific somatic epithelial cell type.

## DISCUSSION

Our objective was to identify the p63^+^/K14^+^ cells that can arise from hES cells. We and others [33–37] had concluded preliminarily that these “hESderK” cells are keratinocytes. Our extensive analysis and comparison with three structurally and developmentally different p63+ epithelial cell types has disclosed that this identification was incorrect. hESderK cells share with keratinocytes, urothelial cells, and tracheobronchial epithelial cells a set of hypermotility, marker expression, and histogenic characteristics in culture. However, hESderK cells differ from these normal somatic cell types by having less stringent growth requirements, extremely limited replicative potential, and a different tissue architecture in organotypic conditions.

The very limited replicative potential of hESderK cells proved to be the consequence of a p16^INK4A^/p14^ARF^ senescence mechanism, apparently identical to that of normal keratinocytes and other p63^+^ epithelial cell types but subject to induction sooner during serial culture than it is in normal epithelial cells. hESderK cells resembled a short lifespan primary keratinocyte line and the primary urothelial and tracheobronchial epithelial cell lines studied here, in that they were not immortalized by TERT expression alone. Transduction to express bmi1, which maintains the normal, repressed state of the p16^INK4A^/p14^ARF^ locus [47–49], greatly extended the lifespan of hESderK cells without changing morphology, growth requirements, or differentiation characteristics. As previously reported for keratinocytes [49], but unlike a result reported for a mammary epithelial cell line [47], bmi1 expression in the epithelial cell types that we studied did not facilitate derepression of endogenous *TERT* to yield immortalized lines but, instead, made cells permissive for TERT immortalization. An advantage of using bmi1+TERT to immortalize epithelial cells is that, unlike E6E7 expression, or the coexpression of dominant-negative mutant p53, p16-resistant mutant cdk4, and TERT that we used previously [23], p53- and pRB-dependent controls remain intact. In situations in which an immortalized line eventually emerges following transduction of keratinocytes to express TERT alone, such lines prove to be variants having genetic or epigenetic alterations of *CDKN2A* resulting in loss of or reduced p16/p14^ARF^ expression [23–25]. Bmi1+TERT expression are well-defined events that obviate selection pressure for mutants. Furthermore, since bmi1 is normally expressed by early passage keratinocytes and other cell types, maintaining expression at levels similar to that of early passage cells should not result in abnormal repression of other genes.

The mechanism triggering p16 expression and senescence appears to be much more sensitive to activation in hESderK cells than in normal somatic p63^+^ epithelial cells. p16 is neither expressed in normal epithelial tissues nor is necessary for their development [28, 29, 31] but is expressed in the epidermis and oral epithelium in vivo in the settings of wound re-epithelialization and neoplastic progression toward squamous cell carcinoma (SCC) [30, 31]. In the latter setting, p16 expression serves as an important tumor suppressor against the formation of SCC. During human development, the definitive epidermis and other stratified squamous epithelia do not form until ∼20 weeks gestation. Complete maturation of mechanisms regulating *CDKN2A*, fine-tuning it for normal repression and ability to be activated in appropriate circumstances, may not occur during the several week period in which hESderK cells form from hES cells in the experimental settings used to date. hESderK cells also do not have the same growth requirements as normal p63^+^ epithelial cells. Unless assisted by E6E7 expression, they did not grow as rapidly as normal p63+ cell types in the feeder/FAD or K-sfm systems and hESderK cells could to grow in serum-supplemented medium without fibroblast feeder cell support and without EGF. They neither form extensive cell-cell junctions nor did stratify in colony centers during growth in the feeder/FAD system, as do normal p63^+^ cell types. This suggests that p63^+^ cells derived experimentally from hES cells either did not have the time or did not have the proper environment to fully develop the growth and differentiation regulatory mechanisms of normal somatic epithelial cells.

Research on p63 has focused on its role in epidermal development, differentiation, and proliferative potential [38, 39, 42, 58] but many other epithelia express p63, and this protein is essential for their development (for example, see refs [41, 59, 60].). An earlier study [19] showed that several nonkeratinocyte epithelial cell types, including urothelial cells, form colonies from single cells, divide rapidly, and can be serially passaged in the feeder/FAD system. Normal tracheobronchial epithelial cells proved here to grow well in this system as well. As reported previously for keratinocytes [20], primary lines of these other cell types grew well in semidefined media, with urothelial cells growing optimally in K-sfm and tracheobronchial epithelial cells in BEGM. The expression of Laminin-332 by hESderK cells and the induction of directional hypermotility by TGFβ or the γ2 chain precursor form of Laminin-332 [30, 31] at first seemed to support their identity as keratinocyte. Our finding that urothelial cells and tracheobronchial epithelial cells also display this response, together with the results of a separate study of human prostate epithelial cells (Wei and Rheinwald, in preparation) now permits us to generalize this as a response of all p63^+^, Laminin-332^+^ cell types.

The morphology, growth requirements, and differentiation characteristics of urothelial and tracheobronchial epithelial cells in culture more closely resembled those of keratinocytes than of hESderK cells. We were most surprised by the similar histogenic behavior and involucrin and K10 expression of all these cell types in organotypic culture. Our results showed that p63^+^ epithelial cell types of endodermal origin, which normally either do not stratify or do not become cornified in vivo, express in culture the stratified squamous epithelial cornified envelope protein involucrin and the epidermoid differentiation-related keratin filament protein K10. The organotypic culture system widely used to evaluate epithelial differentiation was originally optimized to yield a well-differentiated epidermis from cultured epidermal keratinocytes [11, 12]. We found previously that this system results in more epidermal-like suprabasal differentiation by nonepidermal keratinocyte subtypes than those subtypes display in vivo [13]. Involucrin expression and stratified, epidermoid differentiation exhibited by tracheobronchial epithelial cells, which form a ciliated, pseudostratifed, involucrin-negative epithelium in vivo [40], indicate that these cells express an abnormal, squamous metaplastic, differentiation program in culture. Interestingly, the propensity of many epithelial cell types to display this behavior was first detected for rat cells by their formation of cornified envelopes [61] in culture. Increasing the retinoic acid concentration in the organotypic culture medium was insufficient to prevent this squamous metaplastic behavior in our study and in a previous one [13]. Differences among normal epithelial cell types, even among different keratinocyte subtypes (e.g., oral vs. epidermal), with respect to expression of some marker cytokeratin proteins persist during growth in conventional culture [9, 10, 13, 19, 51], but the tendency toward epidermoid differentiation in organotypic culture complicates the precise identification of unknown epithelial cell types such as hESderK cells.

Important future goals of this research are to characterize p63^+^ cell types arising from hES cells and improve culture conditions to obtain completely developed, proliferative somatic epithelial cell types restricted to formation of a single tissue. Previous studies have focused on the H9 and H1 hES cell lines. It will be very important to examine the p63^+^ differentiation potential of other hES lines, because hES lines vary with respect to their abilities to develop into various lineages [62]. The hESderK cell lines we have isolated appear to be an immature stage, or abnormal form, of p63^+^ epithelial development and it remains to be determined whether they are on the pathway to ectodermal or endodermal lineages. Exposure to certain types of fetal mesenchyme may provide specific direction and induce lineage maturation. The morphology and some of the growth characteristics of the hESderK cells are reminiscent of the XB2 murine teratoma-derived, keratinocyte-like cell line, the discovery and early experimentation of which [63] led to the first serial cultivation of normal human keratinocytes [21]. The hESderK/bmi1 line, able to grow progressively but not rapidly in media now available, should prove useful for optimzing media to culture and study precursors and intermediate stages of epithelial development as it occurs naturally or during somatic cell generation from hES or induced pluripotent stem cells.

## DISCLOSURE OF POTENTIAL CONFLICTS OF INTEREST

The authors indicate no potential conflict of interest.
